# Rates of agonism among female primates: a cross-taxon perspective

**DOI:** 10.1093/beheco/art076

**Published:** 2013-08-21

**Authors:** Brandon C. Wheeler, Clara J. Scarry, Andreas Koenig

**Affiliations:** ^a^Cognitive Ethology Laboratory, German Primate Center, 37077 Göttingen, Kellnerweg 4, Germany,; ^b^Courant Research Centre “Evolution of Social Behaviour”, University of Göttingen, 37077 Göttingen, Kellnerweg 6, Germany,; ^c^Department of Anthropology, Stony Brook University, Circle Road, SBS Building S-501, Stony Brook, NY 11794-4364, USA, and; ^d^Interdepartmental Doctoral Program in Anthropological Sciences, Stony Brook University, Circle Road, SBS Building S-501, Stony Brook, NY 11794-4364, USA

**Keywords:** aggression, feeding competition, folivory, frugivory, group size, terrestriality.

## Abstract

Agonism is common in group-living animals, shaping dominance relationships and ultimately impacting individual fitness. Rates of agonism vary considerably among taxa, however, and explaining this variation has been central in ecological models of female social relationships in primates. Early iterations of these models posited a link to diet, with more frequent agonism predicted in frugivorous species due to the presumed greater contestability of fruits relative to other food types. Although some more recent studies have suggested that dietary categories may be poor predictors of contest competition among primates, to date there have been no broad, cross-taxa comparisons of rates of female–female agonism in relation to diet. This study tests whether dietary variables do indeed predict rates of female agonism and further investigates the role of group size (i.e., number of competitors) and substrate use (i.e., degree of arboreality) on the frequency of agonism. Data from 44 wild, unprovisioned groups, including 3 strepsirhine species, 3 platyrrhines, 5 colobines, 10 cercopithecines, and 2 hominoids were analyzed using phylogenetically controlled and uncontrolled methods. Results indicate that diet does not predict agonistic rates, with trends actually being in the opposite direction than predicted for all taxa except cercopithecines. In contrast, agonistic rates are positively associated with group size and possibly degree of terrestriality. Competitor density and perhaps the risk of fighting, thus, appear more important than general diet in predicting agonism among female primates. We discuss the implications of these results for socio-ecological hypotheses.

## Lay summary

It has long been thought that female-female aggression in primates is higher in species that primarily eat fruits than in those that feed more on leaves or insects. Here we test this hypothesis with data from 23 primate species, and show that primates that eat more fruits do not engage in more aggression. Instead, rates of aggression increase with group size and time spent on the ground. Thus, female aggression depends on the density of competitors and the ease or costs of aggression.

## INTRODUCTION

Intraspecific agonistic interactions are a nearly universal consequence of the competition for limited resources that animals face. Especially among animals that live in social groups, regular competition with conspecifics makes agonism a part of daily life (Zinner and Wheeler 2012). The outcome of agonistic interactions are of major evolutionary consequence, given that they determine dominance relationships (Drews 1993), which can ultimately affect an individual’s fitness (Harcourt 1987; Cowlishaw and Dunbar 1991; Keller and Reeve 1994; Frank et al. 1995; Côté and Festa-Bianchet 2001; Say et al. 2001; Majolo et al. 2012; Pusey 2012) due to the greater access of dominants to limited resources, such as food, mates, territory, preferred spatial positions, or social partners (Clutton-Brock 1982; Goss-Custard et al. 1982; Whitten 1983; Thouless 1990; Borries et al. 1991; Krause 1994; Ron et al. 1996; Vogel and Janson 2007; Hirsch 2011). The link between agonistic interactions and access to resources may be direct, as when individuals engage in aggressive competition for access to food (Janson 1985a), or indirect, as when individuals avoid engaging in contests with individuals they perceive as likely to win the interaction (Thouless 1990; Vogel 2005).

In general, agonism is expected primarily in association with high-value resources that—due to their size, depletion time, and spatiotemporal distribution—can be monopolized by a subset of individuals to the exclusion of others (Wrangham 1980; van Schaik 1989; Sterck et al. 1997; Isbell and Young 2002). If these criteria are not met, agonism is not expected to occur. For example, when a given resource is not of sufficiently high value relative to other available resources, the costs of engaging in agonism to gain access to it may exceed the benefits gained by doing so (Enquist and Leimar 1987). Alternatively, the resource may be of high value but have a spatiotemporal distribution that makes agonistic defense impossible or less profitable than alternative strategies (Monaghan and Metcalfe 1985; Janson 1996; Goldberg et al. 2001; Marmet et al. 2012). Variation in the availability, depletion time, and distribution of such resources is, thus, hypothesized to be a major factor explaining the observed variation in the rate at which individuals of a given population interact agonistically (van Schaik 1989; Isbell 1991).

Due to the fact that access to feeding resources is an important factor affecting female reproductive success (Trivers 1972; Emlen and Oring 1977; Wrangham 1979; Lee 1987), the general idea that the distribution of food shapes relationships among females within and between groups has figured prominently in ecological models of female social relationships among primates (Wrangham 1980; van Schaik 1989; Isbell 1991; Sterck et al. 1997; Isbell and Young 2002; Koenig 2002) and other mammals (Clutton-Brock and Janson 2012). According to these models, the occurrence of high-value foods in patches of intermediate size relative to group size and spread is expected to lead to within-group contest competition, that is, rank-related skew in energy gain occurring with or without direct agonistic competition (Janson and van Schaik 1988; Koenig and Borries 2006). In such cases, one or more individuals within a group are able to monopolize access to a food patch to the exclusion of other group members, with access being dependent on the outcome of concurrent or prior agonistic interactions, resulting in higher net energy gain for more dominant individuals. In contrast, when foods are low quality, highly dispersed, or found in patches that are large relative to group size, within-group contest competition is not expected to occur (Koenig 2002). Because of the advantages afforded to winners of agonistic interactions (i.e., more dominant individuals) in the context of contest competition, ecological models of female social relationships predict higher rates of female–female agonistic interactions when food resources are monopolizable, which in turn favors female philopatry and kin-based, despotic dominance relationships among females (van Schaik 1989; Sterck et al. 1997).

Previous research has lent widespread support to the idea that agonism among primate females occurs more frequently during feeding than in nonfeeding contexts (Hill and Okayasu 1995; Sterck and Steenbeek 1997; Cords 2000; Pruetz and Isbell 2000; Range and Noë 2002; Koenig et al. 2004; Koenig and Borries 2006), and that within feeding contexts, agonism is more frequent when foods are more contestable (Janson 1985a; Barton and Whiten 1993; Phillips 1995; Janson 1996; Sterck and Steenbeek 1997; Pruetz and Isbell 2000; Korstjens et al. 2002; Vogel 2005; Chancellor and Isbell 2009b). There is also some support for the more specific contention of the models that, across species, variation in rates of agonism among females can be largely explained by the degree to which individuals rely on clumped food resources, coming primarily from studies of closely related primate species that vary in this aspect of their feeding ecology (Mitchell et al. 1991; Barton et al. 1996). For example, in separate comparisons across baboon (*Papio* spp.) and squirrel monkey (*Saimiri* spp.) taxa, rates of female–female agonism were higher within populations that relied more on foods that occurred in patches that were medium-sized relative to group size and lower in the species that fed more often on foods that were dispersed or occurred in smaller patches (Mitchell et al. 1991; Barton et al. 1996; Boinski et al. 2002).

Unfortunately, very few studies of agonism in primates have included measures of food distribution beyond botanical indices, which may not reflect the contestability of resources on a scale relevant to the study animals, that is, food distribution relative to the size and spread of the group (Koenig and Borries 2006; Vogel et al. 2007; Vogel and Janson 2011; Koenig et al. 2013). In the absence of these critical measures, a broad test of the predicted relationship between rates of agonism and food distribution across primates cannot be conducted. It has been suggested, however, that general dietary categories (frugivory, folivory, insectivory) may reflect the underlying food distribution; whereas fruits are assumed to occur in discrete, high-value patches that can be defended against conspecifics, leaves and insects are often assumed to be relatively lower value and more evenly distributed throughout the environment (Wrangham 1980; van Schaik 1989; Clutton-Brock and Janson 2012). Indeed, some authors have argued that leaves and insects may be so abundant as to constitute a nonlimiting resource (Isbell 1991; Isbell and Young 2002), and a number of studies have indicated that folivores may indeed face reduced feeding competition (Janson and Goldsmith 1995; Steenbeek and van Schaik 2001). It has, thus, been predicted that female–female agonism should be higher in association with feeding on fruits than on either leaves or insects (overview in Snaith and Chapman 2007). Nevertheless, several studies have indicated that immature leaves, which tend to be preferred by folivorous primates over mature leaves (Yeager and Kool 2000), can indeed have limited availability and sometimes occur in discrete patches, potentially eliciting both contest and scramble competition (Koenig et al. 1998; Snaith and Chapman 2007; Sayers 2013), whereas others have shown that increased fruit feeding does not necessarily lead to an increase in within-group contest competition (Heesen et al. 2013) or agonism (Chancellor and Isbell 2009b). Although these studies indicate that broad dietary categories do not always map neatly onto competitive regimes, it is still generally held that they tend to elicit different types and intensities of competition with folivores experiencing, on average, less agonistic competition than frugivores (Clutton-Brock and Janson 2012). However, whether frugivory is indeed associated with higher rates of agonism among female primates remains unknown.

Although consideration of agonism in ecological models of female social relationships have focused exclusively on the effects of food distribution, other factors that have been shown to affect rates of within-group agonistic interactions have received less attention. For example, a number of intraspecific comparisons in primates and other mammals have demonstrated that an increase in group size (and thus competitor density) is associated with an increase in the rate at which individuals interact agonistically with group mates (van Schaik et al. 1983; Janson 1988; Hoogland 1995; Miller 1996; Koenig and Borries 2006). In contrast, it was recently suggested that increasing group size has a negligible effect on feeding competition except at unusually large group sizes (Sussman and Garber 2011), a contention supported by a few studies demonstrating no group size effect on agonism within populations (Risenhoover and Bailey 1985; Chancellor and Isbell 2009a). To date, however, there have been no attempts to examine the effect of group size on agonistic rates across taxa.

Similarly, the degree to which animals feed on terrestrial versus arboreal substrates may significantly affect the rate of agonistic interactions (Hill and Okayasu 1995). On the ground, higher rates might be expected because the complexity of the arboreal environment (e.g., gaps in the canopy) will sometimes limit an individual’s ability to approach a competitor to initiate an interaction. Further, agonism on arboreal substrates may involve additional energetic costs, as well as the added risk of falling from an elevated substrate as a result of the interaction (Broom et al. 2009). Although there are some aspects of terrestriality that could potentially increase the costs of agonism on such substrates (e.g., the increased ease of movement could result in agonism on terrestrial substrates more often leading to physical contact and injury, thus favoring mechanisms to limit agonism), on balance these considerations would seem to suggest that agonism should be more common among more terrestrial species. At present, there have been no systematic studies of the importance of substrate use or group size on agonism, which is likely to have repercussions on the interpretation of these behaviors in regard to models of the ecology of female social relationships.

This study aims to describe variation in rates of female–female agonism in group-living nonhuman primates, including species from each major taxonomic radiation while testing possible predictor variables in a phylogenetically controlled analysis. Specifically, based on assumptions regarding the effect of food types on agonism, we predicted that the frequency of agonistic behavior should increase with increasing amounts of fruits in the diet and decrease with increasing amounts of leaves and/or animal matter in the diet. In addition, we predicted that rates of agonism among females should increase with both female group size and degree of terrestriality.

## METHODS

### Data collection

We undertook an extensive survey of the primate literature published from 1974 to October 2011 to obtain rates of female–female agonistic interactions, defined here as the “number of interactions per adult female focal hour,” for groups of wild, unprovisioned, non-crop-raiding subjects. In addition, unpublished rates of agonism were sought in 2004 and 2012 by contacting individuals who have conducted extensive field research with a given species (see Acknowledgments). Data describing diet, substrate use, and female group size were also obtained in this manner. Altogether the data set consists of 44 groups (or populations; see below) from 23 species including Strepsirhini (3 species), Platyrrhini (3), Cercopithecinae (10), Colobinae (5), and Hominoidea (2) collected at 24 different field sites (see full data set and phylogeny in the Supplementary Material). Although the sample was biased toward Cercopithecinae, and more species from other taxa would have been ideal, this is a result of the fact that—as a group—cercopithecines have been better studied than other taxa (Moore 1984; Strier 1994), especially in terms of female–female social relationships.

### Definitions and data selection

#### Agonism

Comparisons of rates of agonism across taxa are complicated by the fact that the definition of agonism often varies from study to study and what actually constitutes an aggressive or submissive interaction may vary from species to species (Klein 1974). The rates included in this analysis are based on spatial displacements (see definition in Borries et al. 1991), as well as interactions that were considered to be aggressive or submissive for that particular species (i.e., the behaviors considered to be agonistic by the researcher). Problems may, therefore, arise if not all aggressive, and submissive interactions are recognized as such by the observer or if nonagonistic behaviors were misclassified as agonistic. However, because very few studies have tested the function of behaviors or correspondence of aggressive and submissive behaviors in predicting dominance rank (primates: Knox and Sade 1991; Lu et al. 2008; wolves: van Hooff and Wensing 1987), this potential bias could not be taken into account.

Previous work on agonistic rates among primates has included data collected with various different observation methods, assuming weak effects of the different techniques (Sussman et al. 2005). However, this procedure may lead to bias as, for example, time sampling methods will not generate true rates (Koenig et al. 2006; Martin and Bateson 2007), although they may be estimated under certain conditions (Altmann and Wagner 1970). Thus, to avoid bias and to ensure accurate and reliable values of the average number of interactions per individual and hour, we excluded all observations via ad libitum or any time sampling method (Altmann 1974; Martin and Bateson 2007). All agonistic rates used in this analysis were based exclusively on data collected through continuous focal animal sampling of identified females (see also Erhart and Overdorff 2008). We also excluded data collected solely through “all occurrence” methods at the group level (Altmann 1974; Martin and Bateson 2007) because in all but very open environments and with small cohesive groups, the entire group cannot be observed at all times. In addition, while all events of overt aggression may be noticeable in such situations (Asensio et al. 2008), more subtle agonism, particularly facial threats and other nonovert behaviors, will sometimes go unnoticed and lead to an underestimation of rates of agonism.

Ideally, agonistic rates should be calculated and compared for different forms of agonism (displacements, submission, aggression) and for different contexts (feeding, nonfeeding). However, because such data were rarely available, only overall female–female agonistic rates were used without differentiating further (see Discussion). Agonistic rates per adult female per focal observation hour were calculated from the original source or the value given in the source was used. Whenever possible we tried to exclude nonadult females from the calculation, relying on the definition of adulthood by the respective authors. In case of multiple groups from the same species and variable definitions of adulthood, we did not attempt to standardize the age definition. Because competition is believed to be density dependent and driven by the number of competitors within an area or group (Crombie 1947; Nicholson 1954; Janson and van Schaik 1988), in all possible circumstances, agonistic rates for individual groups, rather than species or population averages, were used in the analysis. Rates for 5 populations used in this analysis were averaged over several groups from one site because rates for individual groups were not available. In 4 of these cases, group size differences were minimal (*Propithecus verreauxi*: 2–4 females; *Alouatta pigra*: 2–3 females; *Cercopithecus mitis*: 15–17 females; *P. thomasi*: 2–3 females), but the fifth case involved larger differences in group size (*Macaca fascicularis*: 3–11 individuals; see the Supplementary Material for details and references). Finally, socio-ecological models predict that local ecological conditions affect female relationships (van Schaik 1989; Isbell 1991), suggesting that habitat differences between sites or across seasons may lead to variation in rates of interactions. Thus, whenever possible agonistic rates refer to an average value over a full year (or multiple years). In the absence of such data, we used values if they came from at least one wet and one dry season combined.

#### Diet, substrate use, and group size

All data relating to diet and substrate use came from the same study population as the rates of interactions, although not necessarily from the same study group or the same year. For all groups of a given population, we used either the only available data set or an average for diet and substrate use. Although data for each individual study group would have been ideal, such data were usually not available. The percent of the diet consisting of particular food types was based on percent time feeding on each type. Fruit included both fruits and seeds, leaves also included stems and shoots, and animal matter included both invertebrate and vertebrate prey.

Previous work on agonistic rates has attempted to control for the effect of group size by dividing data for agonism by the number of females (Sussman et al. 2005). Instead, here we chose to test the actual effect by using female group size as predictor or covariate. The number of females in a group was taken from the source for the data on agonistic behavior. The number was averaged if there was variation in this number over the study period or if rates of agonism for multiple groups were pooled (see above). Because in fission–fusion societies, such as in ruffed lemurs, spider monkeys, or chimpanzees, not all members of a group are together most of the time and parties vary in size and composition (Aureli et al. 2008), we used the average number of adult females per party instead of the overall number of females per group, where applicable. In the following the terms “group size” and “party size” will be used interchangeably.

Substrate use, that is degree of arboreality versus terrestriality, was broken down into 3 arbitrary categories: 1) terrestrial, 0–33% arboreal; 2) terrestrial-arboreal, 34–67% arboreal; and 3) arboreal, 68–100% arboreal. If actual percent values for a group were unavailable, we used the categorical classifications given by authors. If those were unavailable, we checked directly with the authors themselves (see Acknowledgments). Hanuman langurs were considered in the terrestrial-arboreal category. Although at this site the langurs are arboreal most of the time (15.7% terrestrial), they are terrestrial during parts of the year (34% in spring) and frequently rest on the ground (Borries and Koenig, unpublished data). The use of the arboreal category for Hanuman langurs did not change the results (data not shown).

### Statistical analyses

All statistical analyses were performed using SPSS 15.0 or R 2.14.0 (R Development Core Team 2011). We performed both phylogentically controlled and across-group (uncontrolled) analyses. Here, we present both analyses because Pagel’s lambda varied considerably across analyses (Freckleton 2009).

We conducted bivariate least square regressions, one-way Anovas (analysis of variance; Sokal and Rohlf 2012), and bivariate phylogenetic generalized least squares (PGLS) regressions (Felsenstein 1988; Orme et al. 2011) to test for the effect of the predictor variables fruit, leaves, animal matter, female group size, and substrate use. The PGLS regressions were performed using the “pgls” function in the caper package (Orme et al. 2011). For each model, we used a maximum-likelihood (ML) approach to simultaneously estimate Pagel’s lambda (Pagel 1999) for the regression parameter(s) and the residual error in the rates of agonism (Revell 2010). The estimate of lambda provides a measure of the degree of the phylogenetic correlation present in the data (Pagel 1999), which can be incorporated into the model by transforming branch lengths to reflect the degree of expected covariance due to phylogeny (Pagel 1999; Freckleton et al. 2002; Orme et al. 2011). Because ML estimations of lambda derived from phylogenies with low numbers of tips may become stuck on a local peak (Freckleton et al. 2002), we checked whether fixing lambda at the upper bound from the 95% confidence interval of lambda provided by the ML estimation affected the results in cases in which the ML estimate of lambda was 0. We used a consensus tree derived from 200 trees downloaded from the 10kTrees project (version 3; Arnold et al. 2010) to generate a phylogeny for all the species in our data set (see Supplementary Figure S1). In this tree, we assigned multiple populations of a single species branch lengths proportional to the geographic distance between them (data not shown in the Supplementary Figure S1). For each lineage, we calculated an estimated rate of change (years per kilometer) by dividing the time since divergence from the nearest sister species available through the 10kTrees project by the geographic distance between the centroids of species ranges as provided in the IUCN Red List of Threatened Species (IUCN 2012).

To test multiple factors simultaneously, we included female group size, substrate use, and amount of fruit in the diet in one multivariate model that incorporated phylogenetic structure. Due to the fact that the different dietary variables are highly correlated and provide measures of the same general variable of interest (i.e., diet), we included amount of fruit as the only dietary variable in the model because it is suggested to be the primary predictor of intragroup agonistic competition (McKenna 1979). Of the remaining variables included in the model, only substrate use and group size show a degree of correlation (terrestriality and group size: Spearman’s ρ = 0.509, *P* = 0.006, *n* = 28; terrestriality and frugivory: ρ = −0.022, *P* = 0.918, *n* = 25; group size and frugivory: *ρ* = −0.052, *P* = 0.799, *n* = 26). Although correlated predictors can sometimes generate problems in identifying the best fit model, the Akaike information criterion (AIC) is considered to be robust in such cases, and it is not normally recommended to exclude correlated variables when they measure different phenomena (Freckleton 2011). We fit the full model including all 3 factors, as well as all subset models, selecting the model with the lowest AIC value (corrected for small sample size, AICc) as the best fit model (Burnham and Anderson 2002). We calculated the evidence ratio (ER; Burnham and Anderson 2002; Symonds and Moussalli 2011) of competing models to assess the likelihood that the best fit model was a better approximation of the underlying process. We used linear mixed-effect modeling in the across-group analysis and PGLS in the phylogenetically controlled model. In addition, to incorporate uncertainty in the positions of taxa and branch lengths, we repeated all analyses across the complete tree block (*n* = 200) and not just the consensus tree. For this analysis, we considered only the ML estimation of lambda.

Data transformations were undertaken to ensure that assumptions of the statistical analyses were met. Rates of agonism were square root transformed, dietary variables were arcsine transformed, and group sizes were log transformed (Sokal and Rohlf 2012). All tests were 2-tailed.

## RESULTS

For this sample of nonhuman primates, we found an overall mean rate of agonism (± standard error [SE]) of 0.61±0.09 SE interactions per female per hour, or slightly more than 1 interaction every 2h (Supplementary Figure S2). The overall range was large, ranging from 0.01 to almost 3 interactions per hour, but most values fell between 0.18 and 0.89 per hour (lower and upper quartiles). Variation in rates of agonism was considerably higher between species than within species (coefficient of variation between species: 87.9%; within species: 47.2%).

**Figure 1:**
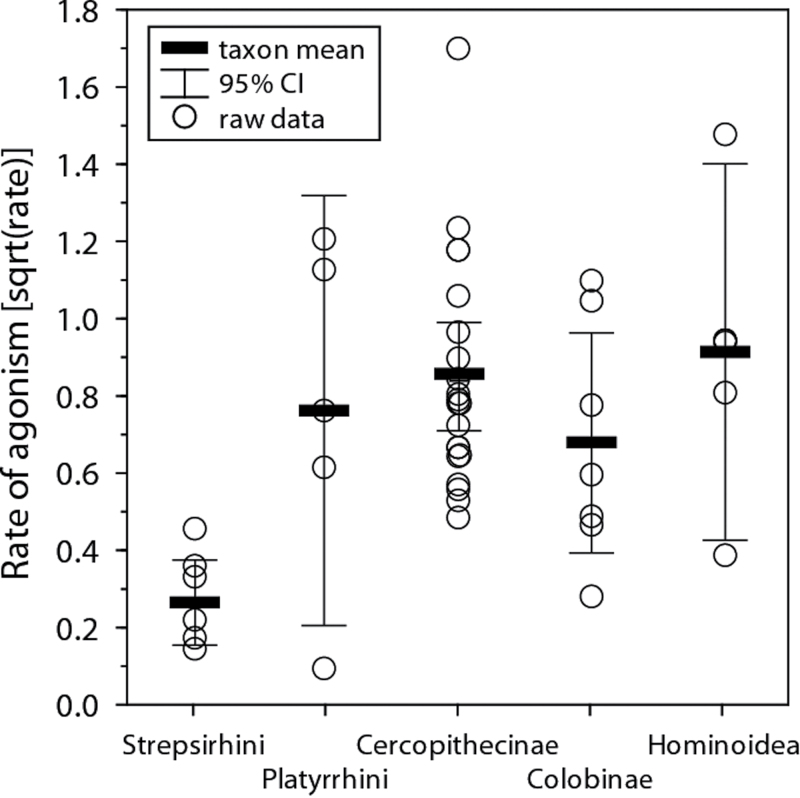
Rates of female–female agonism across major taxonomic groups of nonhuman primates. Data shown are those from the across-group analysis analyzed with a one-way Anova.

When comparing across the major taxonomic groups, overall averages and variation were rather similar, with the exception of low rates of agonism and a small variation for Strepsirhini (one-way Anova: *F*_4,39_ = 5.139, *P* = 0.002; Figure 1), which showed significantly lower rates than both Cercopithecinae and Hominoidea (post hoc Tukey: *P*s < 0.01). After taking phylogeny into account, only the lower rates among Strepsirhini relative to Cercopithecinae remained statistically significant (PGLS: β = −0.566, *t*_26_ = −2.67, *P* = 0.013, *n* = 28, *r*^2^_adj_ = 0.134).

**Figure 2:**
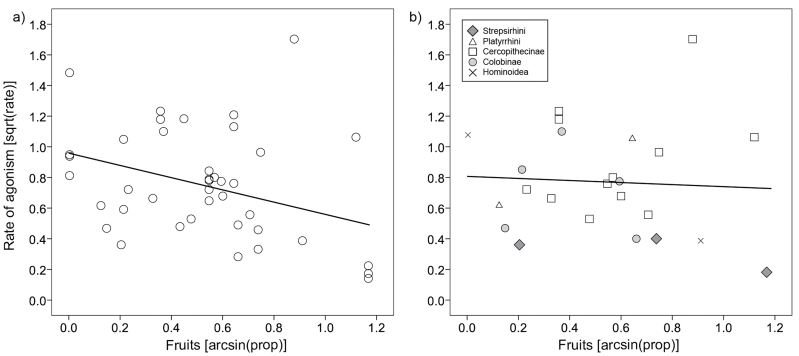
Rates of female–female agonism in relation to the amount of fruits in the diet analyzed by (a) standard statistical methods (least square regression) and (b) phylogenetic methods (PGLS).

The amount of fruits in the diet had a significant effect in the opposite direction than predicted in the across-group analysis, with agonistic rates decreasing with increasing amount of fruits (Pearson’s *r* = –0.366, *P* = 0.019, *n* = 41; Figure 2a). However, the amount of variance explained was low (*r*^2^_adj_ = 0.111). Further, the PGLS regression indicated no influence of the amount of fruits (*β* = −0.026, *t*_22_ = −0.12, *P* = 0.91, *n* = 24, *r*^2^_adj_ < 0.001; Figure 2b) with a moderate but nonsignificant phylogenetic signal (Pagel’s λ = 0.299, 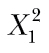 = 1.05, *P* = 0.30) present in the residuals, suggesting that the PGLS analysis is more appropriate than the simple analysis across groups. Inspection of Figure 2b suggests that the effect of fruits on agonistic rates varies across taxonomic groups, so we performed a post hoc analysis examining the interaction between proportion of fruit in the diet and taxonomic group. Among cercopithecine primates, there is a trend toward increasing frugivory being associated with higher rates of female–female agonism (β = 0.884, *t*_22_ = 1.93, *P* = 0.068, *n* = 24; *r*^2^_adj_ = 0.188). The amount of leaves in the diet did not have an effect on the rates of agonism in either the across-group analysis (*r* = 0.058, *P* = 0.742, *n* = 35; *r*^2^_adj_ = −0.027) or in the PGLS regression (ML estimation: β = −0.130, *t*_19_ = −0.57, *P* = 0.58, *n* = 21, *r*^2^_adj_ < 0.001, λ < 0.001; upper bounded lambda: β = −0.041, *t*_19_ = −0.16, *P* = 0.87, *n* = 21, *r*^2^_adj_ < 0.001; λ = 0.678; Supplementary Figure S3). The same was true for the amount of animal matter in the diet (across groups: *r* = 0.131, *P* = 0.453, *n* = 35; *r*^2^_adj_ = −0.013; PGLS regression, ML estimation: β =0.399, *t*_19_ = 0.89, *P* = 0.38, *n* = 21, *r*^2^_adj_ < 0.001, *λ* < 0.001; upper bounded lambda: *β* = −0.15, *t*_19_ = 0.21, *P* = 0.84, *n* = 21, *r*^2^_adj_ < 0.001; Supplementary Figure S4).

**Figure 3:**
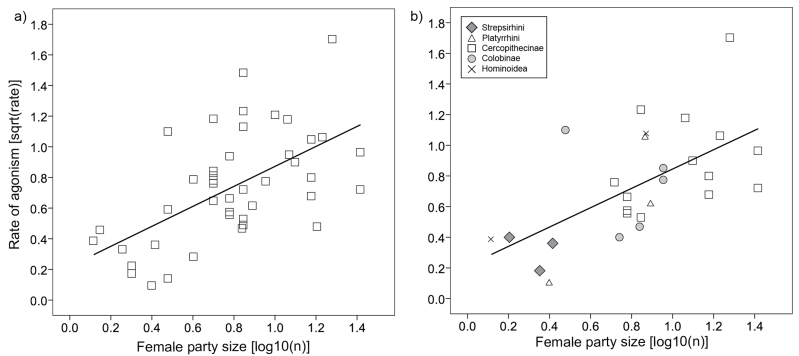
Rates of female–female agonism in relation to female party size (number of females per group or average party size in fission–fusion societies) analyzed by (a) standard statistical methods (least square regression) and (b) phylogenetic methods (PGLS).

Female group size had the expected effect, with the rate of agonism increasing with increasing number of females in a group (least squares regression: *r* = 0.592, *P* < 0.001, *n* = 44; *r*^2^_adj_ = 0.335; Figure 3a). This effect remained significant when controlling for phylogeny both when using the ML estimation of lambda (PGLS: β = 0.631, *t*_25_ = 3.83, *P* < 0.001, *n* = 27, *r*^2^_adj_ = 0.344, λ < 0.001; Figure 3b) and when lambda was fixed using the upper limit of the 95% confidence interval (β = 0.544, *t*_25_ = 2.72, *P* = 0.012, *n* = 27, *λ* = 0.417), although the amount of variance explained was lower in the latter (*r*^2^_adj_ = 0.197). Although significant in all cases, the scatter was rather wide and the amount of variance explained was moderate (<35%).

**Figure 4:**
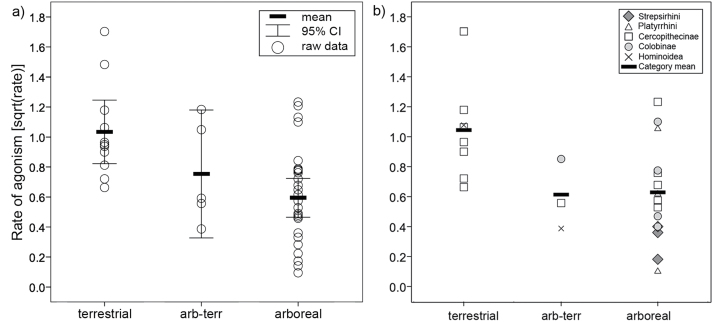
Rates of female–female agonism in relation to substrate use analyzed by (a) standard statistical methods (one-way Anova) and (b) phylogenetic methods (PGLS). See Methods for definitions of substrate use.

Finally, we found a significant effect of substrate use on rates of agonism in the across-group analysis (*F*_2,40_ = 7.123, *P* < 0.001; Figure 4a). As predicted terrestrial groups showed more agonism than arboreal groups (post hoc Tukey: *P* = 0.002), although there was no significant difference between terrestrial and terrestrial-arboreal groups (post hoc Tukey: *P* = 0.259). In the PGLS analysis, we found similar results with terrestrial groups having significantly higher rates of agonism relative to semiarboreal and arboreal taxa (β = 0.421, *t*_24_ = 3.17, *P* = 0.004, *n* = 26, *r*^2^_adj_ = 0.266, λ < 0.001; Figure 4b) or conversely, significantly lower rates among arboreal species relative to all others (β = −0.300, *t*_24_ = –2.23, *P* = 0.035, *n* = 26, *r*^2^_adj_ = 0.137, λ < 0.001). Terrestrial-arboreal groups did not differ significantly from the others (β = −0.194, *t*_26_ = −0.93, *P* = 0.36, *n* = 28, *r*^2^_adj_ < 0.001, λ = 0.343). If the value of lambda is fixed following the upper bounds on the 95% confidence interval provided by ML estimation, however, only the higher rates of agonism among terrestrial species remain statistically significant (terrestrial vs. other: β = 0.327, *t*_24_ = 2.17, *P* = 0.040, *n* = 26, *r*^2^_adj_ = 0.123, λ = 0.592; arboreal vs. other: β = −0.169, *t*_24_ = –1.09, *P* = 0.29, *n* = 26, *r*^2^_adj_ = 0.007, *λ* = 0.685).

**Figure 5:**
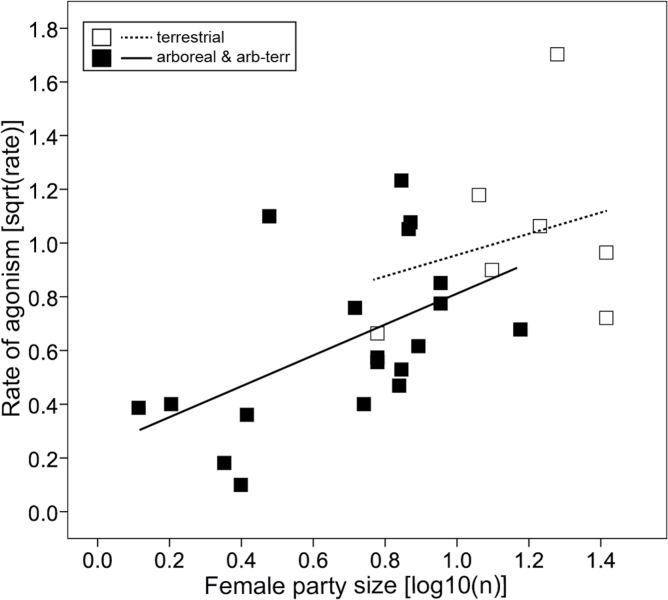
Rates of female–female agonism in relation to female party size (number of females per group or average party size in fission–fusion societies) and substrate use analyzed by phylogenetic methods (PGLS). See Methods for definitions of substrate use. For this figure, the categories arboreal-terrestrial and arboreal were lumped.

In full models including the amount of fruit, number of females, and substrate use, the best subset models never included amount of fruits. In the PGLS analysis (using the upper bounds of the lambda estimate), the best subset model included only the number of females, although models including only substrate use (Δ_AICc_ = 1.63, ER = 2.25) and combining female group size and substrate use (Δ_AICc_ = 0.76, ER = 1.46) are also included within the confidence set of models (see Burnham and Anderson 2002). This result is not an artifact of tree topology, as indicated by the results of multimodel inference across the tree block. For all tree topologies, female group size is the only predictor in the best fit model, and simultaneous estimation of the regression parameters and phylogenetic signal suggests that the relationship between these 2 variables is not phylogenetically constrained (e.g., error in the model is due to measurement error or variable expression: Revell 2010). Nevertheless, this model only slightly outperforms a more complex model that includes both female group size and substrate use (ER = 1.05). Because unnecessary application of phylogenetic regression can produce inaccurate parameter estimates (Revell 2010), we refit the model including all available data points. In the across-group analysis, the best subset model with the lowest AICc value (22.24) included both the number of females and substrate use (i.e., rate of agonism increases with group size and terrestriality; see Figure 5), although the confidence model set also contains a simpler model including only female group size (Δ_AICc_ = 0.70, ER = 1.42).

## DISCUSSION

Rates of agonism among female primates were found to vary considerably within and across taxa, ranging from only a single agonistic interaction per individual every few days in some populations to multiple interactions per hour in others. We found considerable variation in rates of agonism both within and between the major taxonomic groups; the very lowest rates are found only among lemurs and some platyrrhine taxa, and cercopithecines and hominoids tend to be characterized by relatively high rates. In contrast to predictions of the early iterations of the ecological models of primate female social relationships (Wrangham 1980; van Schaik 1989; Isbell 1991), the results of this study do not support the contention that more frugivorous primates tend to experience higher rates of female–female agonism than do more folivorous primates as a result of relaxed feeding competition in the latter (McKenna 1979). First, diet was a weak predictor of agonistic rates relative to the other independent variables considered; dietary variables were not included in the best subset models, suggesting that they do not explain more of the variance in rates of agonism beyond that explained by group size. Second, the direction of the effect of frugivory on agonistic rates differed between taxa; although there was a nonsignificant trend in the predicted direction among cercopithecine primates, the trend was in the opposite direction among noncercopithecines in the phylogenetically controlled analysis, with increased frugivory being related to decreased agonism. In contrast, group size was the best predictor of agonistic rates, while the effect of substrate use was more equivocal. In both cases, the effects were in the predicted directions: agonistic interactions were more common in larger groups and in terrestrial relative to arboreal taxa. Thus, the local density of competitors and perhaps the costs and limitations of agonism in the arboreal milieu appear to better explain variation in rates of agonism among female primates than do broad dietary categories. Nevertheless, 2 caveats should be kept in mind. Although the overall sample size is relatively large, it has been greatly reduced for the phylogenetic analysis of substrate use. Firmer conclusions regarding the effects of substrate use on agonism must await the availability of additional data sets (see also below). In addition, a rather large amount of variation remains unexplained, which may (or may not) relate to variation in ecological conditions. Regardless of the effect of ecological conditions, however, it seems unlikely that the unexplained variance was due to high variance within species, as this was considerably lower than between species variance.

### Diet and agonism

The lack of the predicted relationship between dietary variables and agonistic rates across primates provides perhaps the strongest evidence to date against earlier suggestions that the degree of frugivory versus folivory among primates can serve as a proxy of food contestability and therefore of the type and intensity of within-group feeding competition (McKenna 1979; Wrangham 1980; van Schaik 1989). Although some individual case studies of primates have indeed indicated that, within a given population, fruit feeding engenders more agonism than does leaf-eating (blue monkeys, *C. mitis*: Cords 2000; long-tailed macaques, *M. fascicularis*: Sterck 1995), others have shown no such effect (gray-cheeked mangabeys, *Lophocebus albigena*: Chancellor and Isbell 2009b; Thomas langurs, *P. thomasi*: Sterck 1995). Likewise, some studies of folivorous primates have shown that preferred foliage can indeed be limited, variable in quality, and distributed in such a way that elicits contest competition within groups (Koenig et al. 1998; Snaith and Chapman 2007; see also Barton and Whiten 1993). The results of the current cross-taxa analysis strongly suggest that general dietary categories do not provide a reliable indicator of the type or intensity of feeding competition or a predictor of competitive regimes.

Although suggestive, the absence of a sufficient number of studies documenting variation in energy gain among group members or the occurrence of agonism during feeding versus other behavioral contexts makes it difficult to draw firm conclusions regarding the potential relationship between diet, agonism, and within-group contest competition. It is possible that the lack of an effect of diet on rates of agonism did not result from a lack of a difference in the type and intensity of feeding competition but from nonfrugivorous primates more often competing for resources such as within-group spatial positions (Janson 1990; Rayor and Uetz 1990; Krause 1994; Ron et al. 1996; Hirsch 2011), access to preferred social partners (Seyfarth 1977; Tiddi et al. 2012), or mates (Davies et al. 1996; Kuester and Paul 1996). Indeed, there have been suggestions that unimale–multifemale mating systems are more common among folivorous species (Crockett and Janson 2000) and that such mating systems might lead to more competition among females for a relatively limited resource (Harcourt et al. 1981; Small et al. 1988; Cheney et al. 2012). However, there is, thus, far little reason to suspect that female mating competition is higher in folivorous primates (Huchard and Cowlishaw 2011), although this may warrant further investigation. Likewise, there are no theoretical or empirical grounds on which to expect that females are more likely to compete for access to preferred spatial positions or social partners in these taxa. Nevertheless, we suggest that future studies should compare rates of different forms of agonism (i.e., submission, displacements, and aggression) across contexts to potentially shed light on the specific factors that elicit agonism in primates and other group-living animals (but see Silk 2002 for reasons why agonism might be expected to occur outside of competitive contexts).

Even if a more detailed analysis of agonism across contexts revealed a lack of an effect of food type on agonistic rates, as the current study suggests, it does not necessarily follow that this would indicate a lack of a difference in the strength of within-group contest competition (i.e., rank-related variation in net energy gain) associated with fruit versus leaf eating. Indeed, several studies have shown that subordinate individuals will sometimes suffer reduced food intake due to spatial avoidance of contestable patches occupied by more dominant individuals (Janson 1985b cited in Janson and van Schaik 1988; Thouless 1990; Vogel 2005). Such avoidance behaviors are predicted by game-theoretical models because individuals should be less likely to engage in aggressive interactions if it is known beforehand that they are likely to lose the encounter (Maynard-Smith 1982). Given that stable and despotic dominance relationships are thought to emerge when within-group contest competition is strong (Isbell 1991; Sterck et al. 1997), it is possible that rank-related variation in food intake can be associated with low rates of agonism due to subordinates avoiding active contest of food against individuals that are almost sure to win the interaction. In such a situation, fruits could be more contestable than leaves or animal matter but still engender similar rates of aggression.

Nevertheless, the other forms of agonism considered here (i.e., spontaneous submission and in particular spatial displacements) would likely still occur in association with contestable resources. Indeed, a common strategy used by lower ranking animals to obtain contestable resources is to arrive at the food source prior to the arrival of dominants, who subsequently displace the subordinates (Barta and Giraldeau 1998; Di Bitetti and Janson 2001; Dubuc and Chapais 2007; Hirsch 2007). Further, a recent analysis of rates of agonism in relation to directional consistency in dominance interactions (a measure of despotism) among female anthropoids found a nonsignificant tendency for more despotic groups to be characterized by higher rates of agonism (Koenig et al. 2013). Taken together with the widespread evidence that agonistic behaviors, including aggression, increase in frequency when resources are more contestable (Barton and Whiten 1993; Janson 1985a, 1996; Sterck and Steenbeek 1997; Pruetz and Isbell 2000; Korstjens et al. 2002), the lack of a relationship between diet and agonism strongly suggests that the intensity of within-group contest competition does not increase with increasing frugivory among primates. However, the currently available data do not allow for tests that could explicitly distinguish between this and the alternative explanations discussed above. In order to facilitate more detailed cross-taxa analyses, it would be ideal for future field studies to examine rates of different forms of agonism across different behavioral contexts, integrated with measures of resource distribution that are scaled to group size and spread (Koenig and Borries 2006; Vogel and Janson 2011).

### Substrate use and agonism

The variation in rates of agonism in relation to substrate use matched predictions in some analyses, with more terrestrial species being characterized by more frequent agonistic interactions than more arboreal species but was not significant in others. It is unclear if the significant relationship that exists in some models is an artifact of the positive relationship between terrestriality and group size among primates (Clutton-Brock and Harvey 1977), although the results of the multivariate analyses indicate that the degree of terrestriality may explain some of the variance in agonistic rates beyond that explained by group size. Given the equivocal results and the limited power of the analysis (largely a result of the taxonomically uneven distribution of terrestriality), it is currently difficult to determine if rates of agonism are indeed affected either by the energy and risk associated with engaging in an agonistic interaction when in the trees relative to when on the ground or by the fact that the complexity of the arboreal environment sometimes prevents individuals from approaching group members to engage in an agonistic interaction. More firm conclusions in this regard will require studies of agonism in additional taxa, ideally including noncatarrhine primates that spend considerable time on the ground, as well as the comparison of rates of agonism on arboreal and terrestrial substrates in populations that spend a large proportion of time both in the trees and on the ground (Hill and Okayasu 1995).

If terrestriality does indeed lower the costs and increase the opportunities for agonism, then substrate use may have underappreciated consequences on the social structure of animal groups. Because in many species agonism often occurs between coalitions, such coalition formation may be less likely if an individual has to move quickly through an arboreal environment than if it has only to move along a terrestrial substrate. The likelihood of coalitions in turn can affect the types of dominance hierarchies that develop (Broom et al. 2009), with the potential effect of terrestrial groups being more likely to have nepotistic-based hierarchies than arboreal ones. The results of the current analysis suggests that this possibility warrants further investigation; studies of other animal taxa and incorporation of substrate use in mathematical models of agonistic behavior might be especially insightful in this regard.

It is also possible that the results are affected to some extent by sampling bias, with some agonistic interactions being more likely to be missed by observers of arboreal relative to terrestrial primates due to decreased visibility in the former context. The extent of this bias should be limited by the fact that data on agonism were collected solely via focal sampling, a method that technically should eliminate such biases altogether. However, it cannot be ruled out that rates of subtle forms of agonism, such as facial threats, are consistently underestimated to a greater extent among arboreal primates (Hill and Okayasu 1995), and there seems to be no way to test if this is indeed the case.

### Group size and agonism

Finally, female group size was the best predictor of agonistic rates and was the only variable in the best-fitting subset model. These results conflict with recent suggestions that increasing group size should have only minimal effects on feeding competition (Sussman and Garber 2011) but are predicted by game-theoretical models (Sirot 2000; Dubois et al. 2003) and match a number of individual field and captive studies of primates and other animals (Goss-Custard 1980; Jones 1983; van Schaik et al. 1983; Watts 1985; De Ruiter 1986; Janson 1988; Blumstein et al. 1999; Koenig and Borries 2006). Such trends likely result from the fact that an increase in group size results in an increase in the local density of competitors for limited resources (Janson and van Schaik 1988). That is, as the number of individuals in a group grows, so does the probability that one individual will encounter a second; thus, social interactions in general, and not just agonistic interactions, should be expected to increase with group size.

Furthermore, the intensity of within-group contest competition can be expected to increase with group size without any change in resource distribution, as a consequence of a greater number of individuals competing for access to a given patch (Koenig and Borries 2006). For example, a food patch that can simultaneously accommodate 5 foragers would be expected to elicit contest competition only in groups of 6 or more. Thus, for 2 groups living under identical ecological conditions, rates of agonism may be expected to be higher in larger groups as a result of increased within-group contest, underscoring the importance of incorporating measures of group size and group spread when quantifying resource distribution for the purposes of measuring resource contestability (Koenig and Borries 2006; Vogel and Janson 2011).

## CONCLUSIONS

Although the idea that resource distribution is a critical factor shaping the benefits of agonism and the rates at which it occurs (Janson and van Schaik 1988; van Schaik 1989) is widely supported (Janson 1985a; Mitchell et al. 1991; Barton et al. 1996; Koenig et al. 1998; Pruetz and Isbell 2000; Vogel and Janson 2011), the current results add to the evidence that general dietary categories (i.e., frugivory, folivory, insectivory) are a poor proxy for a given animal’s competitive regime (Koenig et al. 1998; Snaith and Chapman 2007) and suggest that even the generalities still often made in this regard (Clutton-Brock and Janson 2012) may be misleading. Rather rates of within-group female–female agonism are best predicted by group size and to some degree by substrate use.

Although both parameters each explain only a modest amount of the variation in rates of female–female agonism among primates, the results suggest that these factors may, to a certain extent, directly affect the intensity of within-group competition (in the case of group size) or the costs and/or potential of engaging in agonism. Given that higher rates of agonism appear to be associated with more despotic dominance relationships within the major anthropoid clades, although not across anthropoids generally (Koenig et al. 2013), the current results suggest that, all else being equal, increasing group size (e.g., in response to predation risk, between group competition, or infanticide risk: Bertram 1978; Treves and Chapman 1996; Wrangham 1980) and perhaps increasing terrestriality may lead to more despotic relationships among females. Ecological models of female social relationships may, thus, become more predictive by incorporating additional factors such as group size and substrate use. The extent to which the effect of group size on agonism is a result of greater contest competition in large relative to small groups in clumps of equal (absolute) size remains unclear. To this end, it would be important to more often incorporate measures of resource distribution that take group size and spread into account rather than those that include only absolute measures (Koenig and Borries 2006; Vogel and Janson 2011; Koenig et al. 2013). Until such data are available for more species, firm conclusions regarding the degree to which such ecological factors drive agonistic and cooperative relationships among female primates, as well as other group-living species, will remain largely elusive.

## SUPPLEMENTARY MATERIAL

Supplementary material can be found at http://www.beheco.oxfordjournals.org/

## FUNDING

C.J.S. was trained in phylogenetic methods through the AnthroTree Workshop supported by National Science Foundation (BCS-0923791) and NESCent (EF-0905606). C.J.S. was supported by a Graduate Assistantship from the College of Arts and Sciences, Stony Brook University and B.C.W. by a National Science Foundation International Research Fellowship (965074) for a portion of the work associated with this manuscript.

## Supplementary Material

Supplementary Data
