# Supplementary material for: Rates of agonism among female primates: a cross-taxon perspective
Source: Behav Ecol. 2013 Aug 21;24(6):1369–80. doi: 10.1093/beheco/art076 (PMC3796709; doi:10.1093/beheco/art076)
Supplement: Supplementary Data [file supp_24_6_1369__index.html]

Rates of agonism among female primates: a cross-taxon perspective — Rates of agonism among female primates: a cross-taxon perspective — Rates of agonism among female primates: a cross-taxon perspective — Supplementary Data 

# Rates of agonism among female primates: a cross-taxon perspective

## Supplementary Data

Data files

**Files in this Data Supplement:**

- Supplementary Data - Supplementary Data
