# Supplementary material for: Rates of agonism among female primates: a cross-taxon perspective
Source: Behav Ecol. 2013 Aug 21;24(6):1369–80. doi: 10.1093/beheco/art076 (PMC3796709; doi:10.1093/beheco/art076)
Supplement: Supplementary Data [file supp_art076_Electronic_supplementary_material_final.docx]

**Electronic supplementary material**

**Rates of agonism among female primates: a cross-taxon perspective**

Brandon C. Wheeler, Clara J. Scarry, Andreas Koenig


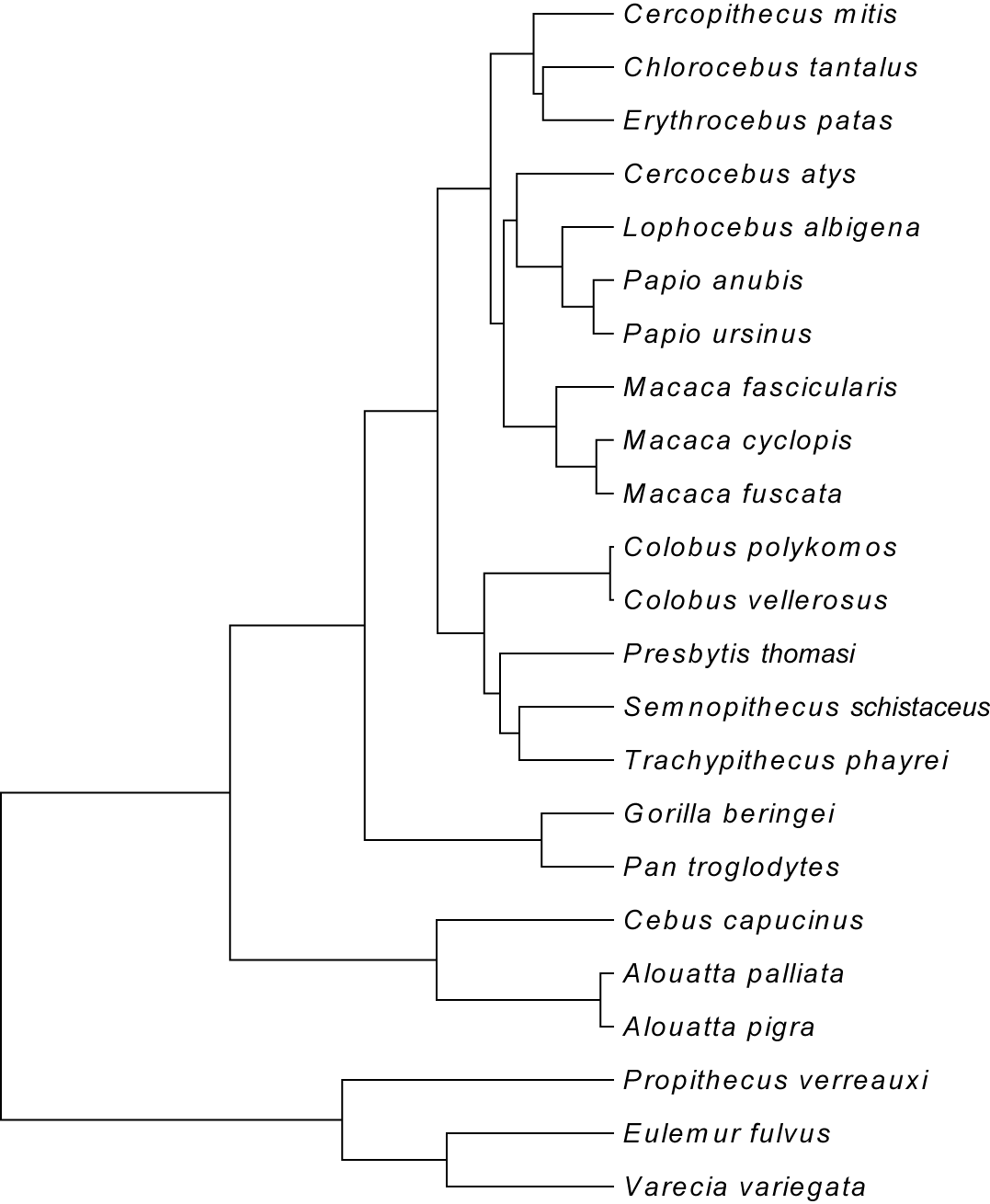


**Fig. S1** Consensus tree used in the study based on a block of 200 trees downloaded from the 10kTrees project (version 3; [Arnold et al. 2010](#_ENREF_2)).


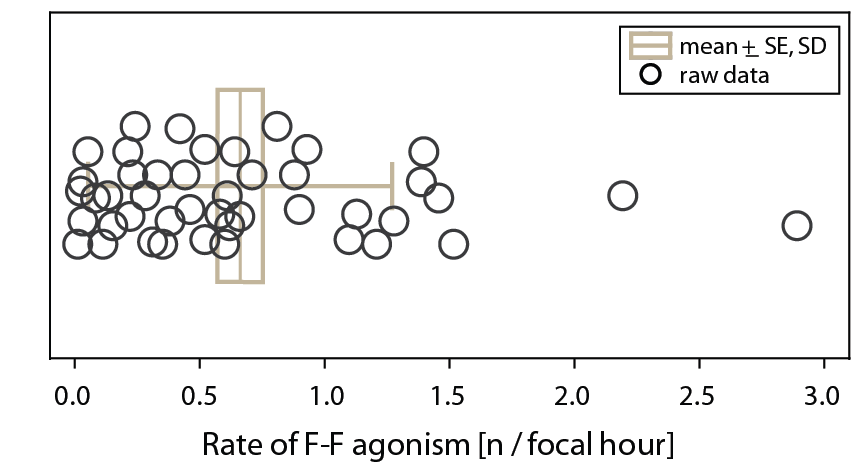


**Fig. S2** Number of female-female agonistic interactions per female focal hour from 44 groups of 23 species of nonhuman primates. The spread on the y-axis is arbitrary to show all of the data points.

**(a)**

**
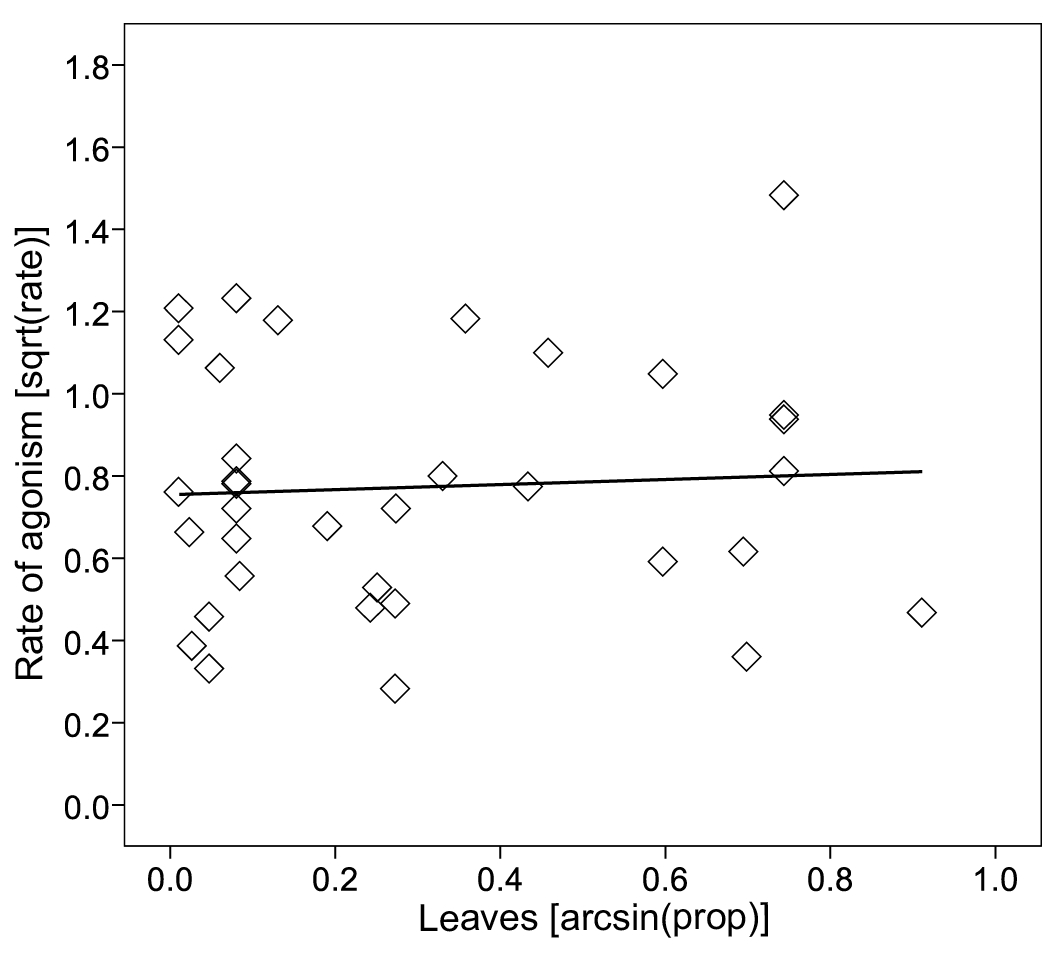
**

**(b)**

**
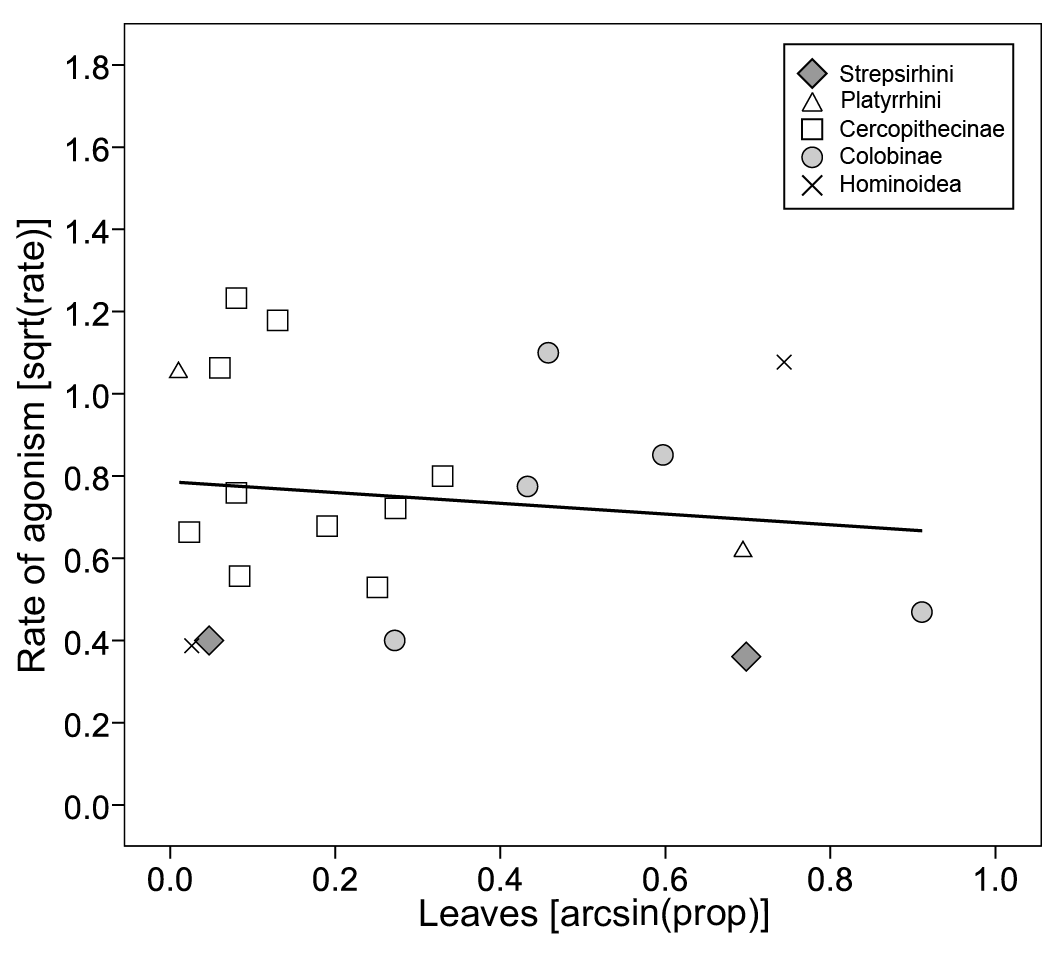
**

**Fig. S3** Rates of female-female agonism in relation to the amount of leaves in the diet analyzed by (**a**) standard statistical methods (least square regression) and (**b**) phylogenetic methods (PGLS).

**(a)**


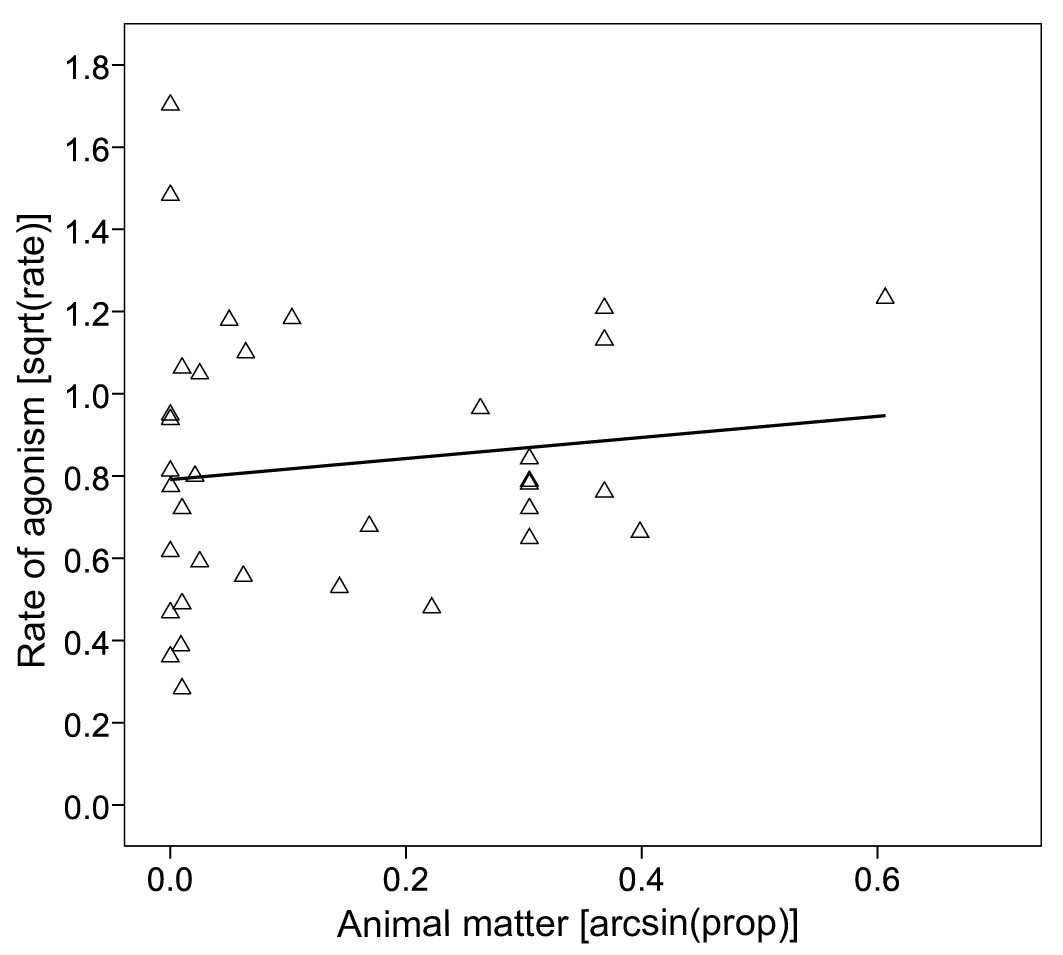


**(b)**


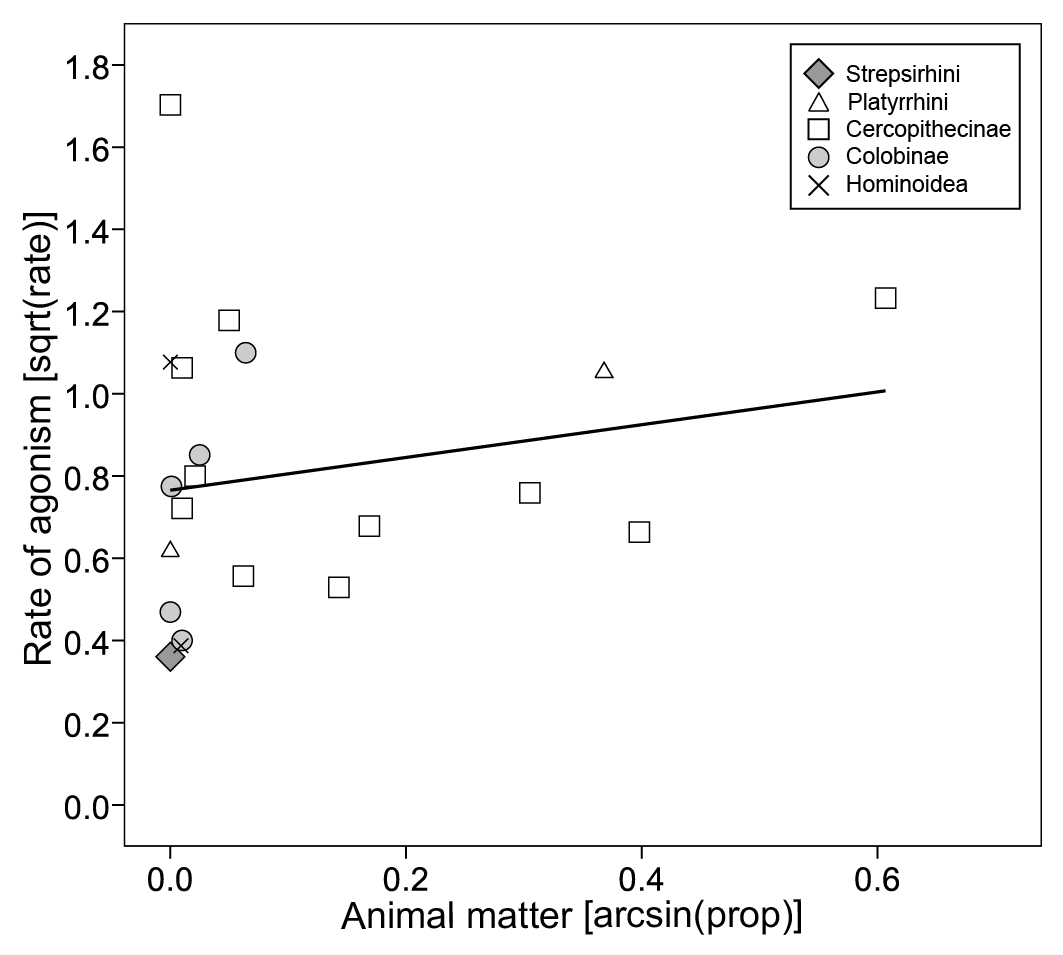


**Fig. S4** Rates of female-female agonism in relation to the amount of animal matter in the diet analyzed by (**a**) standard statistical methods (least square regression) and (**b**) phylogenetic methods (PGLS).

**Table S1.** Data on rates of agonism, diet, substrate use, and female group size used in the analyses.

| **Species†** | **Study site** | **Study group(s)** | **Taxon** | **Agonism/hr** | **% fruit** | **% leaves** | **% animal prey** | **Substrate use^a^** | **♀ group/ party size** | **Refs** |
| --- | --- | --- | --- | --- | --- | --- | --- | --- | --- | --- |
| *Eulemur fulvus* | Kirindy | A | Strepsirhini | 0.05 | 92.0 |  |  | arb | 2.0 | 1 |
| *Eulemur fulvus* | Kirindy | B | Strepsirhini | 0.02 | 92.0 |  |  | arb | 3.0 | 1 |
| *Eulemur fulvus* | Kirindy | J | Strepsirhini | 0.03 | 92.0 |  |  | arb | 2.0 | 1 |
| *Eulemur fulvus* | Kirindy | F | Strepsirhini | 0.03 | 92.0 |  |  | arb | 2.0 | 1 |
| *Propithecus verreauxi*^b^ | Kirindy | A, B, E, H, J | Strepsirhini | 0.13 | 20.3 | 64.3 | 0.0 | arb | 2.6 | 2 |
| *Varecia variegata* | Nosy Mangabe | I | Strepsirhini | 0.11 | 67.3 | 4.7 |  | arb | 1.8 | 3 |
| *Varecia variegata* | Nosy Mangabe | II | Strepsirhini | 0.21 | 67.3 | 4.7 |  | arb | 1.4 | 3 |
| *Alouatta palliata* | La Pacifica | 2 | Platyrrhini | 0.38 | 12.5 | 64.0 | 0.0 | arb | 7.8 | 4 |
| *Alouatta pigra*^b^ | Palenque | Balam, Motiepa | Platyrrhini | 0.01 |  |  |  | arb | 2.5 | 5 |
| *Cebus capucinus* | Santa Rosa | LV | Platyrrhini | 0.58 | 60.0 | 1.0 | 36.0 | arb | 5.0 | 6 |
| *Cebus capucinus* | Santa Rosa | CP | Platyrrhini | 1.28 | 60.0 | 1.0 | 36.0 | arb | 7.0 | 6 |
| *Cebus capucinus* | Santa Rosa | GN | Platyrrhini | 1.46 | 60.0 | 1.0 | 36.0 | arb | 10.0 | 6 |
| *Cercocebus atys* | Taï | not listed | Cercopithecinae | 0.93 | 68.0 |  | 26.0 | terr | 26.0 | 7 |
| *Cercopithecus mitis* | Kakamega | T(w) | Cercopithecinae | 0.46 | 56.5 | 18.9 | 16.8 | arb | 15.0 | 8 |
| *Cercopithecus mitis*^b^ | Kakamega | T(w), G | Cercopithecinae | 0.23 | 42.0 | 24.0 | 22.0 | arb | 16.0 | 9 |
| *Chlorocebus tantalus* | Kala Maloue | S1 | Cercopithecinae | 0.31 | 64.9 | 8.4 | 6.2 | arb/terr | 6.0 | 10 |
| *Erythrocebus patas* | Kala Maloue | KK | Cercopithecinae | 0.44 | 32.2 | 2.3 | 38.8 | terr | 6.0 | 10 |
| *Lophocebus albigena* | Kanyawara | BT1 | Cercopithecinae | 0.71 | 52.0 | 8.0 | 30.0 | arb | 5.0 | 11 |
| *Lophocebus albigena* | Kanyawara | BT2 | Cercopithecinae | 0.62 | 52.0 | 8.0 | 30.0 | arb | 4.0 | 11 |
| *Lophocebus albigena* | Kanyawara | LC | Cercopithecinae | 0.52 | 52.0 | 8.0 | 30.0 | arb | 7.0 | 11 |
| *Lophocebus albigena* | Kanyawara | CC | Cercopithecinae | 0.42 | 52.0 | 8.0 | 30.0 | arb | 5.0 | 11 |
| *Lophocebus albigena* | Kanyawara | MK | Cercopithecinae | 0.61 | 52.0 | 8.0 | 30.0 | arb | 5.0 | 11 |
| *Macaca cyclopis* | Kenting | not listed | Cercopithecinae | 0.33 |  |  |  | arb | 6.0 | 12 |
| *Macaca cyclopis* | Fushan | not listed | Cercopithecinae | 0.28 | 45.9 | 24.8 | 14.3 | arb | 7.0 | 12 |
| *Macaca fascicularis*^b^ | Ketambe | A, H, K, & T | Cercopithecinae | 1.52 | 35.0 | 8.0 | 57.0 | arb | 7.0 | 13 |
| *Macaca fuscata* | Yakushima | P | Cercopithecinae | 1.40 | 43.4 | 35.0 | 10.3 | arb/terr | 5.0 | 14 |
| *Macaca fuscata* | Kinkazan | B(2) | Cercopithecinae | 0.64 | 53.8 | 32.4 | 2.1 |  | 15.0 | 15 |
| *Papio anubis* | Laikipia | not listed | Cercopithecinae | 0.52 | 23.0 | 27.0 | 1.0 | terr | 26.0 | 16 |
| *Papio ursinus* | De Hoop | not listed | Cercopithecinae | 1.39 | 35.0 | 13.0 | 5.0 | terr | 11.5 | 17 |
| *Papio ursinus* | Mkuzi | Mtshopi | Cercopithecinae | 1.13 | 90.0 | 6.0 | 1.0 | terr | 17.0 | 18 |
| *Papio ursinus* | Moremi | not listed | Cercopithecinae | 2.90 | 77.0 |  | 0.0 | terr | 19.0 | 19 |
| *Papio ursinus* | Tsaobis | J, L | Cercopithecinae | 0.81 |  |  |  | terr | 12.5 | 20 |
| *Colobus polykomos* | Taï | Pol(1) | Colobinae | 0.60 | 56.0 | 42.0 | 0.1 | arb | 9.0 | 21 |
| *Colobus vellerosus* | Ghana | not listed | Colobinae | 0.22 | 14.7 | 79.0 | 0.0 | arb | 6.9 | 22 |
| *Presbytis thomasi*^b,c^ | Ketambe | B, J, & M | Colobinae | 1.21 | 36.1 | 44.2 | 6.4 | arb | 3.0 | 23 |
| *Semnopithecus schistaceus* | Ramnagar | O | Colobinae | 1.10 | 21.2 | 56.2 | 2.5 | arb/terr | 15.0 | 24 |
| *Semnopithecus schistaceus* | Ramnagar | A | Colobinae | 0.35 | 21.2 | 56.2 | 2.5 | arb/terr | 3.0 | 24 |
| *Trachypithecus phayrei* | Phu Khieo | PA | Colobinae | 0.24 | 61.3 | 26.9 | 1.0 | arb | 7.0 | 25 |
| *Trachypithecus phayrei* | Phu Khieo | PS | Colobinae | 0.08 | 61.3 | 26.9 | 1.0 | arb | 4.0 | 25 |
| *Gorilla beringei* | Karisoke | 4 | Hominoidea | 0.66 | 0.3 | 67.7 | 0.0 | terr | 5.0 | 26 |
| *Gorilla beringei* | Karisoke | 5 | Hominoidea | 0.90 | 0.3 | 67.7 | 0.0 | terr | 11.7 | 26 |
| *Gorilla beringei* | Karisoke | Nk | Hominoidea | 0.88 | 0.3 | 67.7 | 0.0 | terr | 6.0 | 26 |
| *Gorilla beringei* | Karisoke | Bm | Hominoidea | 2.20 | 0.3 | 67.7 | 0.0 | terr | 7.0 | 26 |
| *Pan troglodytes* | Kibale | Kanyawara | Hominoidea | 0.15 | 79.0 | 2.6 | 0.9 | arb/terr | 1.3 | 27 |

**†** Genus and species names are those currently recognized by Groves ([2001](#_ENREF_15)) and are not necessarily the same as those used by the original authors. **^a.^** arb=arboreal, arb/terr=terrestrial, terr=terrestrial. **^b.^** Rates of interactions are pooled from several groups because data were not available for individual groups. **^c.^** Rate of agonism was calculated by weighting rates of agonism in feeding vs. non-feeding contexts by the time spent in each context**. References: 1.** [Ostner and Kappeler 2004](#_ENREF_35); J. Ostner (unpublished data); **2.** R. Lewis and P. Kappeler (unpublished data); I. Norscia and V. Carrai (unpublished data); [Kappeler and Fichtel 2012](#_ENREF_22); **3.** [Morland 1991](#_ENREF_30); **4.** [Glander 1978](#_ENREF_14); [Zucker and Clarke 1998](#_ENREF_55); **5.** [Van Belle et al. 2011](#_ENREF_49); **6.** Bergstrom 2009; Bergstrom and Fedigan 2010; Rose 1998; **7.** McGraw 1998; Range and Noë 2002; **8**. Cords 1987; Cords 2000; Cords 2002; **9.** [Pazol and Cords 2005](#_ENREF_36); **10.** Nakagawa 2003, 2008; pers. comm.; [Kavanagh 1978](#_ENREF_23); **11.** Chancellor and Isbell 2009; Olupot and Waser 2001; **12.** Su 2001, 2003; Su and Birky (unpublished data); **13.** Reed 1999; van Noordwijk and van Schaik 1987; **14.** Hill 1997; Hill and Okayasu 1995; Kiyono and Hanya (unpublished data); **15.** Agetsuma and Nakagawa 1998; Saito 1996; **16.** Barton 1989; Barton and Whiten 1993; **17.** Barrett et al. 2002; Henzi and Barrett 2003; Hill 1999; **18.** Gaynor 1994; Henzi and Barrett 2003; Ron et al. 1996; **19.** Hamilton et al. 1978; Silk et al. 1999; Silk et al. (unpublished data); **20.** [Huchard and Cowlishaw 2011](#_ENREF_21); **21.** Korstjens 2001; Korstjens et al. 2002; **22.** [Saj and Sicotte 2007](#_ENREF_42); E. Wikberg (unpublished data); **23.** Sterck 1995; Sterck and Steenbeek 1997; **24.** Koenig and Borries 2001; Koenig and Borries 2006; Nikolei 2003; A. Koenig (unpublished data); **25.** [Koenig et al. 2004](#_ENREF_26); A. Koenig, C. Borries, E. Larney, and A. Lu (unpublished data); **26.** Tuttle and Watts 1985; Watts 1984; Watts 1994; **27.** Bean 1999; Wrangham et al. 1996; Wrangham et al. 1992.

**References for Electronic Supplementary Material**

Agetsuma N, Nakagawa N. 1998. Effects of habitat differences on feeding behaviors of Japanese monkeys: comparisons between Yakushima and Kinkazan. Primates 39:275-289.

Arnold C, Matthews LJ, Nunn CL. 2010. The 10k trees website: a new online resource for primate phylogeny. Evol Anthropol 19:114-118.

Barrett L, Gaynor D, Henzi SP. 2002. A dynamic interaction between aggression and grooming reciprocity among female chacma baboons. Anim Behav 63:1047-1053.

Barton RA. 1989. Foraging Strategies, Diet, and Competition in Olive Baboons [Ph.D. thesis]. St. Andrews: University of St. Andrews.

Barton RA, Whiten A. 1993. Feeding competition among female olive baboons, *Papio anubis*. Anim Behav 46:777-789.

Bean A. 1999. Ecology of sex differences in great ape foraging. In: Comparative Primate Socioecology. Cambridge: Cambridge Univ Press; 339-362.

Bergstrom ML. 2009. Dominance Among Female White-Faced Capuchins (*Cebus capucinus*) at Santa Rosa National Park, Costa Rica [Master's thesis]. Calgary: University of Calgary.

Bergstrom ML, Fedigan LM. 2010. Dominance among female white-faced capuchin monkeys (*Cebus capucinus*): hierarchical linearity, nepotism, strength and stability. Behaviour 147:899-931.

Chancellor RL, Isbell LA. 2009. Female grooming markets in a population of gray-cheeked mangabeys (*Lophocebus albigena*). Behav Ecol 20:79-86.

Cords M. 1987. Mixed-species association of *Cercopithecus* monkeys in the Kakamega forest, Kenya. University of California Publications in Zoology 117:1-109.

Cords M. 2000. Agonistic and affiliative relationships in a blue monkey group. In: Old World Monkeys (Whitehead P, Jolly C, eds). Cambridge: Cambridge University Press; 453-479.

Cords M. 2002. Friendship among adult female blue monkeys (*Cercopithecus mitis*). Behaviour 139:291-314.

Gaynor D. 1994. Foraging and feeding behavior of chacma baboons in a woodland habitat [PhD thesis]. Durban: University of Natal.

Glander KE. 1978. Howling monkey feeding behavior and plant secondary compounds: a study of strategies. In: The Ecology of Arboreal Folivores (Montgomery G, ed). Washington, D.C.: Smithsonian Institute Press; 561-574.

Groves CP. 2001. Primate Taxonomy. Washington, DC: Smithsonian Institution Press.

Hamilton WJ, Buskirk RE, Buskirk WH. 1978. Omnivory and utilization of food resources by chacma baboons, *Papio ursinus*. Am Nat 112:911-924.

Henzi P, Barrett L. 2003. Evolutionary ecology, sexual conflict, and behavioral differentiation among baboon populations. Evol Anthropol 12:217-230.

Hill DA. 1997. Seasonal variation in the feeding behavior and diet of Japanese macaques (*Macaca fuscata yakui*) in lowland forest of Yakushima. Am J Primatol 43:305-322.

Hill DA, Okayasu N. 1995. Absence of youngest ascendency in the dominance relations of sisters in wild Japanese macaques (*Macaca fuscata yakui*). Behaviour 132:367-379.

Hill RA. 1999. Determinants of Time Budgets in Baboons: Implications for Cross-Populational Models of Baboon Socioecology [PhD thesis]. Liverpool: University of Liverpool.

Huchard E, Cowlishaw G. 2011. Female-female aggression around mating: an extra cost of sociality in a multimale primate society. Behav Ecol 22:1003-1011.

Kappeler PM, Fichtel C. 2012. A 15-year perspective on the social organization and life history of Sifaka in Kirindy Forest. In: Long-Term Field Studies of Primates (Kappeler PM, Watts DP, eds). Heidelberg: Springer Verlag; 101-121.

Kavanagh M. 1978. Diet and Feeding-Behavior of *Cercopithecus aethiops tantalus*. Folia Primatol 30:30-63.

Koenig A, Borries C. 2001. Socioecology of Hanuman langurs: the story of their success. Evol Anthropol 10:122-137.

Koenig A, Borries C. 2006. The predictive power of socioecological models: a reconsideration of resource characteristics, agonism, and dominance hierarchies. In: Feeding Ecology in Apes and Other Primates: Ecological, Physical and Behavioral Aspects (Hohmann G, Robbins MM, Boesch C, eds). New York: Cambridge University Press; 263-284.

Koenig A, Larney E, Lu A, Borries C. 2004. Agonistic behavior and dominance relationships in female Phayre’s leaf monkeys – prelimary results. Am J Primatol 64:351-357.

Korstjens AH. 2001. The mob, the secret sorority, and the phantoms: an analysis of the socio-ecological strategies of the three colobines of Taï [Ph.D. thesis]. Utrecht: Utrecht University.

Korstjens AH, Sterck EHM, Noë R. 2002. How adaptive or phylogenetically inert is primate social behaviour? A test with two sympatric colobines. Behaviour 139:203-225.

McGraw WS. 1998. Comparative locomotion and habitat use of six monkeys in the Taï Forest, Ivory Coast. Am J Phys Anthropol 105:493–510.

Morland HS. 1991. Social organization and ecology of black and white ruffed lemurs (*Varecia variegata variegata*) in lowland rain forest, Nosy Mangabe, Madagascar [PhD thesis]. New Haven: Yale University.

Nakagawa N. 2003. Difference in food selection between patas monkeys (*Erythrocebus patas*) and tantalus monkeys (*Cercopithecus aethiops tantalus*) in Kala Maloue National Park, Cameroon, in relation to nutrient content. Primates 44:3-11.

Nakagawa N. 2008. Despotic wild patas monkeys (*Erythrocebus patas*) in Kala Maloue, Cameroon. Am J Primatol 70:238-246.

Nikolei J. 2003. Lokomotionsökologie adulter Hanuman-Languren (*Semnopithecus entellus*) in einem saisonalen Waldhabitat in Ramnagar, Südnepal. Osnabrück: Der Andere Verlag.

Olupot W, Waser PM. 2001. Activity patterns, habitat use and mortality risks of mangabey males living outside social groups. Anim Behav 61:1227-1235.

Ostner J, Kappeler P. 2004. Male life history and the unusual adult sex ratios of redfronted lemur, *Eulemur fulvus rufus*, groups. Anim Behav 67:249-259.

Pazol K, Cords M. 2005. Seasonal variation in feeding behavior, competition and female social relationships in a forest dwelling guenon, the blue monkey (*Cercopithecus mitis stuhlmanni*), in the Kakamega Forest, Kenya. Behav Ecol Sociobiol 58:566-577.

Range F, Noë R. 2002. Familiarity and dominance relations among female sooty mangabeys in the Taï National Park. Am J Primatol 56:137-153.

Reed KE. 1999. Population density of primates in communities: differences in community structure. In: Primate Communities (Fleagle JG, Janson CH, Reed KE, eds). Cambridge: Cambridge University Press; 116-140.

Ron T, Henzi SP, Motro U. 1996. Do female chacma baboons compete for a safe spatial position in a southern woodland habitat? Behaviour 133:475-490.

Rose LM. 1998. Behavioral ecology of white-faced capuchins (*Cebus capucinus*) in Costa Rica [Ph.D. thesis]. St. Luis: Washington University.

Saito C. 1996. Dominance and feeding success in female Japanese macaques, *Macaca fuscata*: Effects of food patch size and inter-patch distance. Anim Behav 51:967-980.

Saj TL, Sicotte P. 2007. Scramble competition among *Colobus vellerosus* at Boabeng-Fiema, Ghana. Int J Primatol 28:337-355.

Silk JB, Seyfarth RM, Cheney DL. 1999. The structure of social relationships among female savanna baboons in Moremi Reserve, Botswana. Behaviour 136:679-703.

Sterck EHM. 1995. Females, foods and fights. A socioecological comparison of the sympatric Thomas langur and long-tailed macaque [PhD thesis]. Utrecht: Utrecht University.

Sterck EHM, Steenbeek R. 1997. Female dominance relationships and food competition in the sympatric Thomas langur and long-tailed macaque. Behaviour 134:749-774.

Su H-H. 2001. The feeding competition among females in a Taiwanese macaque group at Fushan Forest, Taiwan. Am J Primatol 57 S1:72.

Su H-H. 2003. Acquirement of social ranks of females in one group of Taiwanese macaques (*Macaca cyclopis*) at Fushan Experimental Forest, Taiwan. Am J Phys Anthropol S36:203.

Tuttle RH, Watts DP. 1985. The positional behavior and adaptive complexes of *Pan gorilla*. In: Primate Morphology, Locomotor Analysis, and Human Bipedalism (Kondo S, ed). Tokyo: University of Tokyo Press; 261-288.

Van Belle S, Estrada A, Strier KB. 2011. Insights into social relationships among female black howler monkeys *Alouatta pigra* at Palenque National Park, Mexico. Curr Zool 57:1-7.

van Noordwijk MA, van Schaik CP. 1987. Competition among female long-tailed macaques, *Macaca fascicularis*. Anim Behav 35:577-589.

Watts DP. 1984. Diet and composition of mountain gorills diets in the central Virungas. Am J Primatol 7:323-356.

Watts DP. 1994. Agonistic relationships between female mountain gorillas (*Gorilla gorilla beringei*). Behav Ecol Sociobiol 34:347-358.

Wrangham RW, Chapman CA, Clark-Arcadi AP, Isabirye-Basute G. 1996. Social ecology of Kanyawara chimpanzees: implications for understanding the costs of great ape groups. In: Great Ape Societies (McGrew WC, Marchant LF, Nishida T, eds). Cambridge: Cambridge University Press; 45-57.

Wrangham RW, Clark AP, Isabirye-Basuta G. 1992. Female social relationships and social organization of Kibale Forest chimpanzees. In: Topics in Primatology Vol 1: Human Origins (T. Nishida WCM, P. Marler, M. Pickford & F.B.M. de Waal, ed). Tokyo: University of Tokyo Press; 81-98.

Zucker EL, Clarke MR. 1998. Agonistic and affiliative relationships of adult female howlers (*Alouatta palliata*) in Costa Rica over a 4-year period. Int J Primatol 19:433-449.
